# Acceptability of minimal invasive tissue sampling (MITS) for stillbirths in Eastern Uganda

**DOI:** 10.1371/journal.pone.0334548

**Published:** 2025-10-13

**Authors:** Martin Chebet, Kathy Burgoine, Joseph Rujumba, Noela Regina Akwi Okalany, Peter Olupot-Olupot, Thorkild Tylleskär, Andrew D. Weeks, Agnes Napyo, David Mukunya, Ingunn Marie S. Engebretsen

**Affiliations:** 1 Department of Paediatrics and Child Health, Busitema University Faculty of Health Sciences, Mbale, Uganda; 2 Centre for International Health, Department of Global Public Health and Primary Care, University of Bergen, Bergen, Norway; 3 Mbale Regional Referral Hospital, Mbale, Uganda; 4 Department of Paediatrics and Child Health, Makerere University College of Health Sciences, Kampala, Uganda; 5 Department of Community and Public Health, Busitema University Faculty of Health Sciences, Mbale, Uganda; 6 Mbale Clinical Research Institute, Mbale, Uganda; 7 Sanyu Research Unit, Department of Women’s and Children’s Health, University of Liverpool, Liverpool, United Kingdom; 8 Department of Nursing, Kabale University, Kabale, Uganda; Aga Khan University Hospital, PAKISTAN

## Abstract

**Background:**

In sub-Saharan Africa, stillbirth rates remain high. To design effective interventions to reduce stillbirths, accurate determination of their aetiology is important. Conventional autopsy for accurate confirmation of cause is not acceptable or feasible in several societies in sub-Saharan Africa; minimal invasive tissue sampling (MITS), is a recently developed, less invasive alternative. In this study, we explored the acceptability of MITS in the community and among healthcare workers in Uganda to guide the future implementation.

**Methods:**

A qualitative study was done among community members and healthcare workers in Mbale in Eastern Uganda. We undertook in-depth interviews and focus group discussions in English or local languages. Interviews were audio-recorded, transcribed as necessary prior to formal content analysis. The themes were organised using NVivo software and presented according to Sekhon’s theoretical framework.

**Results:**

Overall, participants preferred the idea of MITS to conventional autopsy because of the perception that it was fast, maintained the facial appearance and kept the body intact. It was thought that the procedure would improve the detection of the cause of stillbirths, which in turn would help to prevent future stillbirths. It would also resolve conflicts in the community between community members or the women and the healthcare workers about the cause of a stillbirth. It was suggested that some community members may not approve of MITS because of their religious beliefs; the fear that the body parts may be extracted and stolen for witchcraft or organ donation; and a lack of trust in the healthcare system. To implement the procedure, it was suggested that extensive community sensitization should be done, space limitations in healthcare facilities overcome, healthcare workers should be trained and limited human resource should be addressed.

**Conclusion:**

The implementation of MITS in Mbale, Eastern Uganda, is likely to be acceptable given sufficient training and sensitisation.

## Background

Despite reductions in under-five, infant and neonatal mortality globally, stillbirth rates have remained high. In 2021, there were about 1.9 million stillbirths globally [1], corresponding to a global stillbirth rate at 14 per 1000 total births. Sub-Saharan Africa is disproportionally affected since it has less than a third of the world’s live births, but almost half of the stillbirths [1]. Most countries in sub-Saharan Africa are estimated to have more than 20 stillbirths per 1000 total births [2]. Uganda was estimated to have a stillbirth rate of 15.2 per 1000 total births in 2021 [1].

To effectively reduce the rate of stillbirths, the causes must be better appreciated. Following routine laboratory and imaging investigations, the cause of stillbirths may still not be determined in about 1 in 4 cases, more so among antepartum stillbirths [3]. In such patients, autopsy is the gold standard to assess the cause. In many low-and-middle-income countries (LMICs), conventional autopsy is not readily available because of lack of qualified pathologists. In Uganda, most pathologists are employed in healthcare facilities around the capital city Kampala. Findings from the Ministry of Health indicate that only two of the thirteen regional referral hospitals have a pathologist [4]. This means the ability to perform conventional autopsies in healthcare facilities outside of Kampala is limited. Even when a conventional autopsy is possible, it is known to be unacceptable in some cultures within Uganda, with up to nearly two-thirds of people declining conventional autopsies in Uganda [5]. Reasons identified include a fear of delaying the burial process, failure to see the importance for an autopsy, satisfaction with the clinical diagnosis and the fear of disfigurement of the body [5,6].

As an alternative to conventional autopsy, World Health Organization (WHO) recommends verbal autopsy [7,8]. In verbal autopsy, the cause of death is determined by analysis of data captured through a structured interview with caregivers or next of kin of the deceased [7]. One major limitation with verbal autopsy is accuracy which is usually low in situations where the signs and symptoms of the disease were non-specific, when there is delay between the death and verbal autopsy and when the death occurred in the community [9]. There is therefore need for a more reliable, culturally sensitive and accessible procedures.

Minimal Invasive Tissue Sampling (MITS), specifically designed for low-resource settings, was developed as an alternative to mitigate some of the challenges with conventional autopsy and verbal autopsy [10,11]. MITS is a relatively new procedure that involves careful physical examination of the body to detect any abnormalities, collection of blood and cerebrospinal fluid, taking off swabs from the rectum and oropharyngeal surfaces, and taking lung, liver, heart and brain biopsies using 16G (1.65 mm outer diameter) biopsy needles [12]. The MITS can be performed by a trained technician (who does not have to be a pathologist) close to the place where the death occurred, for example, in a side room of a ward. Non-physician healthcare workers, such as nurses, can be trained to competently perform this procedure [12]. This procedure has been investigated as a tool to determine cause of death in both the paediatric [15,16] and adult populations [12]. Studies have shown that the procedure can accurately determine the cause of death in resource limited settings [10,13]. In a study done in Mozambique, MITS and conventional autopsy confirmed the cause of death in 84% and 98% of deaths respectively [13]. In a study in Kazakhstan, MITS was concordant with conventional autopsy in 83.3% of cause of deaths among stillbirths and neonates [14]. It also determined cause of death in 90.7% of stillbirths in South Africa [15].

Because of the limited availability and poor uptake of conventional autopsy in Uganda, it is important to find an alternative solution to determine the causes of stillbirths. We hypothesised that since MITS, when compared to conventional autopsy, is minimally invasive, faster, and sufficiently accurate, it is likely to be more acceptable in communities where conventional autopsies is unacceptable [16–19]. Data about use or acceptability of MITS in Uganda is scarce. In this study, we explored the acceptability of MITS in the community population and among healthcare workers in Mbale in Eastern Uganda, to guide the future implementation of this technology.

## Methods

The methods and results of this article are written in accordance with the consolidated criteria for reporting qualitative research (COREQ) guidelines [20].

### Study design

This was qualitative descriptive study conducted between 5th October 2023 and 11th May 2024. The study was part of a larger project on stillbirths in Eastern Uganda that included quantitative and qualitative studies surrounding stillbirths in Mbale and Budaka districts, eastern Uganda. The qualitative part of the project involved exploring the beliefs and practices around stillbirths, community perceptions of the causes of stillbirths, health care experiences and social support among women who experienced a stillbirth in Eastern Uganda. The work on community perceptions has been published [21]. The quantitative component included an epidemiological study [22] and microbiological component (ongoing).

### Study setting

The study was done in Mbale District in Eastern Uganda, located about 220 kilometres North-East of Kampala. The study involved both community participants and health workers. The health workers were selected from Busiu Health Centre IV and Nakaloke Health Centre III and Mbale Regional Referral Hospital (MRRH), Mbale Eastern Uganda. MRRH is a tertary hospital that serves fourteen districts in Eastern Uganda. Busiu Health Centre and Nakaloke Health Centre are about 20 and 10 kilometres from MRRH respectively. MRRH experiences between 30–50 stillbirths per month, while the two health centres experience around 4 stillbirths per month. The community participants were selected from areas surrounding Busiu Health Centre IV and Nakaloke Health Centre III. Mbale is one of the six districts predominantly inhabited by the Bagisu, (or Gisu or Bamasaba), one of the largest ethnic groups in Uganda, with an estimated population of around 2 million people. The participants of this study mainly resided in rural areas, whilst a few of them resided in a peri-urban setting, the outskirts of Mbale City. The predominant language spoken is Lumasaba, although Luganda is also spoken by the urban and peri-urban dwellers.

### Study population

The community participants included male and female residents over twenty years old from the study areas who had any childbirth experience, women and their close relatives with a history of stillbirth, cultural leaders, religious leaders and community health workers (CHWs). The age limit of at least twenty years was set by the authors to increase the chances of childbirth experience among potential participants approached. The healthcare workers were from the three health facilities and should have had at least one year experience working with women giving birth.

### Recruitment of participants

All the participants were purposively selected so as to acquire rich, relevant, and diverse information related to the acceptability of MITS. The CHWs, community and religious leaders were selected with the help of the subcounty leaders. The residents and the women with a history of stillbirth were selected with the help of the CHWs. The community participants were selected from within a 10 km radius of Busiu and Nakaloke Health Centres respectively. The healthcare workers were selected with the help of the health unit in-charge. The healthcare workers for each healthcare facility were contacted from the list of names provided by the in-charge of the facility through physical visits or telephone contacts until the required number had been interviewed. If the number for each facility was reached before the list was exhausted, the remaining healthcare workers were not contacted.

### Data collection

We conducted six focus group discussions (FGDs) and 30 in-depth interviews (IDIs). The IDIs were done with community and religious leaders and the women with a history of stillbirths. We conducted one FGD for each of the following groups: (1) women residents aged 20–35 years, (2) women residents above 35 years, (3) male residents aged 20–45 years, (4) male residents above 45 years, (5) community health workers (CHWs), and (6) a group composed exclusively of Muslims (both men and women).We had not initially planned to do a Muslim-only FGD, but we decided to do it when it emerged after analysis of the first FGD and IDIs that they hold unique beliefs about death and stillbirths. Each FGD had 6–8 participants. Characteristics of the participants are given in Table 1.

**Table 1:** Overview of constructs based on Sekhon’s theoretical framework of acceptability, subthemes and codes.

Construct	Subthemes	Codes	Informant
**Themes in line with Sekhon’s theoretical framework of acceptability**			
Affective attitude	Religious beliefs	• Baby’s length of life on earth is pre-determined by God • It is like questioning God’s authority	Community members
	Fear of witchcraft	• Fear that the tissue extracted may be used for witchcraft	Community members
	Lack of trust in the healthcare system	• Women do not utilise the healthcare facility services	Community members
Burden	Space limitations	• Use existing space • Create new space by partitioning some rooms • Improvise furniture	Health workers
	Heavy workload	• Devise ways of working amidst heavy work load • Since stillbirths are rare, health workers can still do MITS amidst heavy workload	Health workers
	Extensive sensitization	• Community outreaches • Use of community health workers • Use platforms such as antenatal clinics	Both health workers and community members
Ethicality	Improving knowledge about the causes of stillbirths within the community	• Help health workers confirm cause when in doubt • Prevent future stillbirth in the same mother • Prevent future stillbirths in other women • Improves quality of care among health workers	Both community members and health workers
	Reduces conflict in the community	• Reduces blame game between the health workers and the women • Reduces accusations about witchcraft	Community members
	It is prohibited to cause harm to a dead body	• Islamic religion believe that a dead body feels pain • Even when they are washing a dead body, they use warm water, not cold and not hot so as not to hurt the body • MITS is less painful than conventional autopsy • MITS can be permitted in Islam as *Dhalula* because of the medical benefits	Community members
	Body is left intact	• The spirit of the stillborn baby is not maimed • The spirit of the stillborn baby will not haunt	Community members
Intervention coherence	Awareness about how causes of stillbirth are made in health facilities.	• Laboratory test • Physical examination • Tissue cutting	Community members
	Awareness about how the MITS procedure is done.	• Removal of body tissue • Removal of organs of the body • Done in big hospitals only • Done in adults only	Community members
	Awareness about reasons for doing MITS.	• To determine the cause of stillbirth • Selling organs for donation	Community members
Intervention effectiveness	Faster than conventional autopsy	• Avoids perceived delay to release the stillborn baby to the relatives • Allows immediate burial to take place	Community members and Health workers
	Face is not disfigured	• The face can still be recognisable • You can be sure that the health workers have given you the right body	Community members
Self-efficacy	Train health workers	• No health workers are trained to perform MITS	Health workers

The interviews and discussions were conducted by the first author (MC), a male paediatrician trained in qualitative research and four trained female research midwives, who all held a bachelor’s of midwifery and had prior experience in qualitative research. The interviewers were fluent in both the local languages and English, thus facilitating data collection in a language that the participants were conversant with.

Data were collected using an interview guide for IDIs and focus group guide for focus group discussions, translated into the local languages (Lumasaba and Luganda). The questions and prompts were pretested to ensure that they were clear and captured the information it was intended to capture. After identification, potential participants were invited to participate in the study through a phone call or by a physical visit. Except one healthcare worker who was on leave, all participants agreed to take part in the study and no repeat interview or FGD was done. The venue, date and time of the interview was agreed upon between the participants and the researchers. The IDIs were done at the health facilities, at the homes or in the workplaces of the participants. Privacy of the participants and confidentiality of data was ensured. The IDI involved one participant and one researcher and were done in a private room where no one else was present. The FGDs took place in a room where only the participants and researchers were present. At least two researchers were usually present for each FGD, one led the discussion and the other took notes. All FGDs took place at the healthcare facility. Two FGDs (CHWs and Muslims) were conducted in Luganda and the rest were in Lumasaba. Most IDIs were conducted in Lumasaba with a few in English and Luganda.

The introduction of MITS in the interviews and discussions happened after the participants already had discussed their views about stillbirth and practices surrounding it facilitating a more natural introduction to the topic which otherwise could be stressful. Participants were encouraged to ask questions whenever they wanted. We then asked the participants about their perceptions about MITS. The assessment of the hypothetical acceptability of the MITS procedure was done after a brief (10 minutes) description to the participants of how the procedure of MITS is done by the aid of photographs showing the procedures that have been previously published and are available online [23] (Supporting information S1 Fig). We did not simulate the MITS procedure.

The IDIs took between 30 and 50 minutes while the FGDs took between one and a half to two hours. Field notes were taken to capture any non-verbal cues during the interview. Data were collected until no major new themes emerged. The transcripts were not returned to the participants for comments.

### Data management

The audio recordings were transcribed verbatim and those in the local languages were translated to English before being transcribed as the written local languages are irregularly used. The translation from local language to English was done by people fluent in the two languages and with experience in translation of similar research data for several years. The audio recordings and transcripts were deidentified and stored as password protected files. Consent forms and other paper documents were stored in a locked cabinet.

### Data analysis

The transcripts were imported and analysed using Nvivo version 14, R 1.7.2. Thematic content analysis was done following the steps described by Braun and Clarke [24]. We read the texts multiple times to familiarise ourselves with the data. Each transcript was coded by two coders, MC and AN. The two coders reviewed the codes that they generated to come up with the resultant codes that were used for the final analysis. After identifying patterns among the codes, themes were inductively generated to categorise the codes. No themes were identified or developed before data collection. We reviewed the themes against the codes and against the text to ensure that they accurately depicted what the data presented. This iterative process including re-naming and re-arrangement of themes was repeated until the final list of themes and codes was arrived at.

### The theoretical framework

The interview and discussion guides were planned as an independent data collection, and during the initial conductive data analysis it was decided to use Sekhon’s theoretical framework of acceptability to present the findings [25] for its good fit. The framework was developed based on reviews of existing reviews and theories for the purpose of assessing the multiple constructs of acceptability of healthcare interventions. It can be used pre-intervention, concurrent with an intervention or post-intervention. It can also be used to assess the perceptions of the recipients or the implementors or deliverers of the intervention. It has seven constructs: *affective attitude, burden, opportunity cost, ethicality, intervention coherence, self-efficacy* and *perceived effectiveness. Affective attitude* is about how an individual feels about taking part in an intervention. *Burden* is the perceived amount of effort that is required to participate in the intervention for example time, expense and cognitive effort needed. *Opportunity cost* refer to the extent to which benefits, profits, or values must be given up to engage in an intervention. *Ethicality* is the extent to which the intervention has good fit with an individual’s value system. *Intervention coherence* is the extent to which the participant understands the intervention, and how the intervention works. *Self-efficacy* is the participant’s confidence that they can perform the behaviour(s) required to participate in the intervention. *Perceived effectiveness* is the extent to which the intervention is perceived as likely to achieve its purpose.

### Reflexivity

The first author (MC), although not originally from the study area has worked in a health facility within the study area for more than 15 years. Among the research midwives, only one was a Gisu by ethnicity who grew up from the same community while the rest were from different ethnic groups in central Uganda. The researchers, predominantly coming from a biomedical tradition, having had training and experience in qualitative research, were aware of their own assumptions and prejudices. They made a conscious attempt to limit the interference from their own assumptions with the interviews and analysis stages knowing that complete objectivity is not possible. The fact that the interviewers were healthcare workers may have prevented some participants from fully expressing their non-medical opinions about MITS. This is because they may provide answers they think the healthcare worker wants to hear. Furthermore, they may have seen the healthcare worker as an authority figure making them feel intimidated or fear to be judged causing them to withhold or alter their true beliefs. We tried to mitigate this by assuring the participants before every interview that it is important to understand the community perception about MITS so that culturally appropriate decisions can be considered in future. This study was done as part of the PhD research for the first author who was a PhD candidate at the time of the research. There was no relationship established between the interviewers and the participants prior to the commencement of study.

### Ethics

The study was done in accordance with the Declaration of Helsinki. Ethical approval was obtained from Uganda National Council of Science and Technology (reference number HS3112ES) and Busitema University Faculty of Health Sciences Research and Ethics committee # BUFHS-2022-18) and Regional Committee for Medical and Health Research Ethics, Norway (REK West #278845). All the participants were above 18 years of age. Written informed consent was obtained from each participant before enrolment. The midwives also counselled women who needed psychological support. Women who needed medical care or advanced psychological support were referred to appropriate health professionals. The audio recording will be destroyed 1 year after the study has been completed.

## Results

### Participant characteristics

We conducted 30 IDIs which included 12 with women who had experienced a stillbirth, 4 with men whose spouses had experienced a stillbirth, 2 with men whose daughter had experienced a stillbirth, 6 with healthcare workers (4 midwives, 2 doctors), 4 with religious leaders, one with a cultural leader, and one with a traditional birth attendant. Out of all the participants of IDIs, 24 were between 20 and 45 years of age, 18 were female, 17 had 0–7 years and 11 had 8–13 years of formal education, 21 were Gisu by tribe, and 25 were Christian. Among these participants, 16 had 5 or more children and 21 had a history of a stillbirth.

We also conducted six FGDs. The total number of participants of FGDs was 44 out of whom, 26 were female, 19 were aged between 20 and 29 years and 22 were aged between 30 and 60 years, 27 had up to 7 years and 15 had 8–13 years of formal education. Among the participants of FGDs, 32 were Gisu by ethnic group, 30 were Christian and 23 had history of a stillbirth.

### Emotional reaction to viewing the pictures of MITS procedure

Most participants were comfortable viewing the pictures and suggested them being shown to the community during future sensitisation activities so that they can understand the way the procedure is done. A few participants expressed discomfort viewing the pictures.

*“These pictures will not bring problems. It is good that these pictures reach the community [during sensitisations], so that people can see them and appreciate how the procedure is done.”* (Participant 02, FGD CHW)

### The themes

Six out of seven constructs based on Sekhon’s theoretical framework of acceptability were consistent with the themes identified in this study. An overview is presented in Table 1 followed by a description supported with quotes and narrations.

### Affective attitude

Affective attitude in this study refers to how the participants felt about the procedure being done on stillborn infants in their community. Religious beliefs, the fear that the tissue extracted may be used for witchcraft and lack of trust in the healthcare system responsible for the MITS procedure resulted in limited interest for MITS.

Muslims mentioned that they were unlikely to accept the procedure because they believed that when someone dies, it is the will of God. It was therefore perceived that trying to find out the cause, is actually questioning God’s authority. Members from Christian and Islamic faiths expressed deterministic views about God’s will concerning life and death of a human. However, Christians did not perceive that determining the cause of death through MITS was equivalent to questioning God’s authority. Muslims believed that God determined the kind of life the unborn baby will live and for how long their life on earth will be.

*“When a baby dies in Islam, we assume it is the will of Allah. And Allah will give you something better. So, if you try finding out the cause, it is like you are trying to question Allah’s authority. It’s Allah that knows. Actually, when a baby makes 4 months in the womb, Allah gives them a life contract to sign that will determine the things they will do, the people they will meet in life and how long they will live. That is the time the baby starts to live according to Islam. And that is why before 4 months of [gestation], we are supposed to pray that Allah may choose a good soul to put in the baby in your womb.”* (Participant 8, FGD Muslim)

As it was perceived that God influenced stillbirth, and it was irreversible, this influenced the bereavement. Some mentioned that losing a dear one is like milk which has poured, it can never be recovered, however much you cry.

It was also reported that it was a common belief among people in the community that the body parts of a stillbirth can be used for witchcraft. So, if people realised that some parts have been taken off, they may think it is the mother who has done it, and she might be mistaken for offering the tissue to witchcraft practitioners to be used for witchcraft. In fear of this, the mother or other caretakers may decline the procedure.

*“They might think that you, the mother, are the one who has done it [cut tissue away from the dead baby]. They would think that this woman has been a witchdoctor. She wanted to kill the baby. Some may say that the mother connived with the health workers [to take away the samples for witchcraft].”* (Participant 4 IDI, Woman with history of stillbirth)

A few participants mentioned that some women may not accept MITS because they do not believe in the healthcare system: they do not attend antenatal care, they give birth from home, and do not immunise their babies. This was in contrast with users of the healthcare system that were more likely to accept the procedures.

*“... in my area there are women who never go to antenatal when they conceive, they give birth from home and they do not immunize children until they are adults. So, for such a person they cannot accept at all to take off tissues when the baby is dead for examination. However, there are those who go for antenatal care and give birth in the hospital, these might accept. Because they would wish to know why that child died.”* (Participant 19, FGD young women)

### Burden

Burden in this study refers to the perceived amount of effort in terms of time, preliminary activities and cognitive effort required to introduce the procedure into the community. The perceptions of both the community members (potentially receiving the procedure) and the health workers (potentially delivering the procedure) were considered.

The healthcare workers pointed out the need to identify a space for the MITS procedure. Although most healthcare workers reported space shortages in their facilities, innovative approaches such as use of the already existing procedure rooms, partitioning some large rooms to create space and improvising furniture were suggested to accommodate MITS future implementation.

*“We need the instruments, okay the room is there but then we need infrastructure, yes, we have the screen, the table, a chair and then we need to train the health workers.”* (Participant 03, IDI health worker)

The healthcare workers also commented on the need to devise ways of handling the excessive workload that healthcare workers already are facing, which would be exacerbated by this new procedure.

The health workers mentioned that despite the heavy workload they face, it would still be possible for them to perform MITS. Most of them also believed that their colleagues would be willing to perform MITS if trained.

*“I don’t think this can be an issue [referring to heavy workload] in our facility given that the cases [stillbirths] are not so many, there could be one in a week, one in two weeks, one in a month, so to me it’s not an added burden of responsibility really.”* (Participant 01 IDI, healthcare worker)

The healthcare workers suggested that to improve the uptake, the procedure should be done immediately after the stillbirth. This is because most caretakers would want to take away the body immediately. Any delay would make them anxious.

*“You may need to do this [perform the procedure immediately after birth], because these mothers leave [the health facility] immediately after losing their babies especially during the night*.” (Participant 06, healthcare worker)

The community members and healthcare workers also emphasized the need to do extensive sensitization in the community before implementing the procedure. Most participants (both healthcare workers and the community members) felt that it would be necessary for the healthcare workers to sensitize the community members through public sensitization using radio and community outreaches for example. The more educated and/or exposed people were perceived to be more likely to accept the procedure, but others could be more hesitant.

*“You know in our community we have two kinds of people, there are the educated,...and those who are totally the opposite [uneducated]. So, the educated may easily understand, if I explain to them [the educated], we are doing the procedure because we want to know what caused it [the stillbirth] so that next time you may prevent it, they will understand but the other ones [the uneducated], it will be very difficult [to explain the rationale of the procedure until they understand]. Remember you need time [to explain to them] yet they usually want to rush and go and bury”.* (Participant 05 IDI, healthcare worker)

Other avenues of sensitization suggested by healthcare workers and other participants include the use of CHWs to sensitize the community members, women during antenatal care attendance and other informal routes. It was perceived that CHWs are more likely to be accepted because they are part of the community.

*“It would also be helpful to teach the CHWs so that they can also pass the information among the members of the community. They can spread this information in the community in addition to the mothers coming for ante natal care. Or there are some men who drink alcohol so the CHWs who also drink can pass on the information to them in the village drinking places. When the husband comes back home, he will tell the woman and the woman comes and gets this information.”* (Participant 5, FGD CHW)

Others suggested targeted community campaigns with pregnant women and village leaders could help improve the uptake of the procedure within the community. This could be done through community outreaches or in conjunction with other community health programmes such as immunisation outreaches.

*“If time comes and this method is put into practice, you should consider going to the communities to educate the LC1s [village leader]) who will also in turn teach their members in the villages. Secondly, health workers who come to the villages for immunization can also come along with those pictures and the members in the community keep looking through them so that we can get more information about what will be done (in case of stillbirth).”* (Participant 15, FGD men)

### Ethicality

Ethicality refers to whether the procedure is compatible with the values held by the community. MITS was regarded as a way of improving knowledge about the causes of stillbirths within the community and an important way of preventing future stillbirths. The healthcare workers felt that healthcare workers would welcome the procedure because they were usually faced with difficult situations where the cause of death is not known, and MITS was seen as a possible solution.

*“So, we always review [perinatal death audit], so people are always puzzled [about the cause], about what happened to some stillbirths. You even fail to write the cause of a foetal death. There are cases where you really fail to write what could have happened. So, if there is something which can answer those questions, it would be a good one.”* (Participant 02 IDI, health worker)

Some stillbirths were said to recur in the same mother. Knowing the cause of a stillbirth, could help to plan for appropriate treatment for the mother, hence preventing future stillbirths. The need to know the cause was emphasised for women who have had recurrent stillbirths. Knowing the cause was also said to help other women, other than the affected mother to avoid stillbirths.

*“Although the baby is already dead and the procedure will not bring the baby back to life, I think it is good to find the cause so that others don’t die of the same cause.”* (Participant 1 IDI, religious leader)

Some participants believed that if the cause of death is in doubt, it should be confirmed by MITS because it will improve accountability. This is because if it is found that it is the poor healthcare or negligence from the healthcare workers, then the healthcare workers will be compelled to improve on their care.

*“I see that this is good and let it start immediately and they should not delay so that we know what is going on [causing the deaths]. When it starts immediately deaths will not be many because the health workers will be more careful when managing these women because they will know that if this child dies there will be litigation against them so they will work well, and deaths will be few.”* (Participant 24, FGD young women)

Accusations and disagreements were said to be common between healthcare workers and the women for the cause of death. Healthcare workers were commonly cited to blame the women for their delay to report for medical attention as the cause of stillbirth, while women frequently blamed the healthcare workers for the poor care during their delivery. The MITS procedure was seen as an opportunity to determine the true cause of death, that could reduce these disagreements.

*“Me I think it would be good to know what caused the baby to be born dead. Maybe the illness was from you the mother. Even if the baby has died, when you are told, you can begin the treatment.”* (Participant 41, FGD older women)

Similarly, several women, men and CHWs mentioned that when there was uncertainty about the cause of death, the MITS would be important to confirm the cause. It was reported that if the community suspected that a community member was practising witchcraft to cause stillbirth, he or she may be harmed, sometimes beaten to death or ostracised. Furthermore, it was suggested that confirmation of the cause through MITS by the health worker would reduce speculation that a death may have occurred due to witchcraft.

*“I think using this method of picking samples using the needle to try and know the cause will help us greatly and reduce the wrangles within the community where people accuse others of bewitching them.”* (Participant 18, FGD men)

Among the Muslim community, *physical trauma* was said to be prohibited on a dead body because they believe the body still feels pain. For example, if a mother died giving birth, to avoid inflicting pain on the body, she was buried without removing the baby, provided it was confirmed that the baby was also dead.

*“You see this method, its good because people undergo a lot of pain in the old method where they cut the abdomen, change the body to another side and cut again. If health workers can only use the syringe and prick the person, I see this is a good method.”* (Participant 3, IDI religious leader)

Some Muslims mentioned that even if their religion prohibits cutting of the body, there are some exceptions to the rule if it is for medical reasons. They believed that if the procedure prevented other women from going through the painful experience of a stillbirth in the future, it could be allowed, as saving other future lives overrides some religious values.

*“If you said that they are going to cut the baby and open it up, I would not allow because of my religious beliefs. But if it is just pricking for investigation to find out the cause of death and to save others, I will allow. I believe even for us, so much research was done to make sure we grow up and not die. So, I would let it happen to save another (life) in the future. Because I believe after us, there should be others.” (*Participant 09, FGD Muslim)

*Dhalula* was term used by Muslims to refer to situations when a practice that is usually prohibited in Islam is instead permitted to take place because of the medical or other life benefits that may be associated with it. However, some Muslims mentioned that even if *Dhalula* existed, MITS should not be done among Muslims because by pricking the body, you are hurting it.

*“But there’s something called Dhalula. Something that is inevitable. For example, you are not allowed to pray with an image or photo. But we pray with our identity cards and they have photos. We even pray with money in our pockets [yet they have images that would otherwise be prohibited]. So, this is called Dhalula.”* (Participant 13, FGD Muslim)

*“It is not allowed in Islam because even if it is just bathing the dead body with warm water, you have to first pour it on yourself to make sure it is not so hot and will not burn the dead person. So, what about the pricks? We believe it would be hurting the body.”* (Participant 10, FGD Muslim)

Some also believed that after death, the body ascends to heaven, and one needs to have their body intact for heavenly life. Some related removing the body tissues or organs to maiming someone. For example, they mentioned that if one’s tongue was cut off and buried without it, their spirit would not be able to speak. In the life after death, it was said that the person, just like in the life before death, needs all the organs of their body to live a normal life. They wondered how the organs removed will reach heaven to reunite with the owner of the body.

*“So now we cannot say that we can cut off someone`s eye or let us cut off the nose to perform tests or let us cut off the neck or the thigh. How does it look when you go with half body to the after-life when they have just cut you? I wonder where we are heading to.”* (Participant 10, FGD Muslim)

Some believed that since the body parts are not replaced into the body after removal, the spirit of the dead person may come back to haunt the relatives.

*“I think this new method is very good and if people are educated about it, they will surely support it. This is because people had a lot of fear for post-mortem especially when they would tell them to take their beloved people since they would open up the body and it would be buried empty without organs. So, the spirit of the dead would come back and disturb them. But this new method will be supported by many. The old method [conventional autopsy] has been scaring people a lot and they did not support it at all.”* (Participant 19, FGD men)

### Intervention coherence

Intervention coherence is the degree to which the participant understands what is involved during the procedure and how it works. In this study, we considered the awareness of participants about how stillbirth causes are confirmed in the health facilities, their awareness about how the MITS procedure is done and the reasons for doing it.

Most respondents knew about some of the ways that health workers confirm the cause of stillbirth. They mentioned laboratory testing of blood, urine and other body fluids, tissue *cutting* and physical examination as common ways health workers could confirm the cause of stillbirth. From this, it was mentioned that one could tell if the mother had an illness that may have caused the stillbirth.

*“The health workers can confirm what killed the baby when they send you to the laboratory to do the necessary tests. They test urine or blood or any other thing and after that they tell you what caused the death of the baby. When they write, the health worker reads and tells you what killed the baby so that you can also know.”* (Participant 24, FGD young women)

Some mentioned that health workers can know what caused the stillbirth by examining the dead baby.

*“What I think is, if the baby dies in the womb and is born, it would require the health workers to examine the baby who has died to know the cause.”* (Participant 26, FGD young women)

Few participants from the community mentioned that the health workers could *cut off* tissue from the dead baby and examine them to find out the cause.

*“They can cut some tissue or remove blood and they test and know what caused the baby’s death. That can also be helpful. Currently, when the health workers want to find out more about you, they send you to the lab, they can take off sputum, urine or blood and test. After the tests they can tell the illness that caused the baby’s death.”* (Participant 46, FGD older women)

None of the health workers or other participants had heard about MITS.

*“No, it’s the first time [to hear about it] and no one is trained and we are not doing it. I knew of the fine needle biopsy, is it almost the same?”* (Participant 05, health worker)

Most of the community members mentioned that they had not heard about MITS. Some mentioned that they had a close relative die and autopsy was done on them. Some mentioned that it was done in big hospitals only and in adults but not for children or stillborn babies.

*“I know that this is done on dead bodies, they open up their bodies. I personally have lost my brothers and they did post-mortem on them. They opened up their bodies.”* (Participant 13, FGD Muslim)

The participants had varied perceptions about the processes involved in an autopsy. Some mentioned that it involves cutting away organs from the body for example removing the brain while others were not sure what the procedure involves.

*“Those things can be done for example if a person was beaten, they would take to police, then to doctor to operate and do autopsy then they get the cause of death but to be done in stillbirths I have not heard.”* (Participant 01 IDI, religious leader)

Participants had multiple perceptions about the reasons for doing an autopsy. While most of the attendants knew why autopsy is done, a few mentioned that they did not know. Most of them mentioned that autopsy is done to find out the cause of death in people whose cause of death is in doubt.

*“Yes, I have heard about this, and it also applies to adults, it is true when a person has died, they have to confirm the cause of death. Questions will arise; “this person has been here, what has caused their death right now?” Definitely they have to take them to Mbale Regional Referral Hospital for autopsy. Sometimes they may be investigating whether they poisoned him or whether it is true that that the deceased has been sick? or he/she was knocked accidentally? Or what indeed has caused his/her death? Was it pressure [hypertension.] My children did not get a conventional autopsy performed on them. Or maybe let me say that none of my relatives or myself thought about taking them for a conventional autopsy, for the health worker or doctor to finally inform me that,” your child or children died because of this illness, after performing an autopsy”. No, it was not like that. We left the health unit straight to bury them.”* (Participant 07 IDI, woman with history of stillbirth)

A few respondents mentioned that conventional autopsy was done to remove organs from the dead body and sold out for organ transplant without the knowledge of the relatives of the deceased. This was said to be a reason for most families declining autopsy.

*“And from what we hear, there are government programs, that one may die, but when they have organs that are still fine and could help others that are still alive. So, we suspect that maybe some of these organs may be removed and given to those that are alive.”* (Participant 8, FGD Muslim)

### Perceived effectiveness

Perceived effectiveness is the extent to which the procedure was perceived to achieve the purpose. In addition to the intended purpose of confirming the cause of death, MITS was perceived to have other inherent advantages such as being faster than conventional autopsy, its minimal invasiveness that leads to no disfigurement of the face and keeping the body and its organs intact as desired by most people in the community. The support of the procedure was mostly done in comparison with conventional autopsy.

Most respondents expressed the desire for confirmation of the cause of death to be done faster, preferably before the burial. They mentioned that with the conventional method samples are taken for analysis and the results sent back when it too late.

*“I think this method will be good even in the community because the current method takes long and the way they cut the body; it might scare you to even touch the body of your loved one. Secondly, this method will bring about knowing quickly what killed the person thereby reducing unnecessary words/rumours in the community which is not the case with the current method.”* (Participant 17, FGD men)

The health workers perceived that since MITS takes relatively short time to perform, it is more likely to be acceptable than conventional autopsy. But a requirement would be that the MITS procedures would be done as soon as soon as possible as it was a universal practice for relatives to take the body as soon after the baby is born to prepare for the burial and associated practices and rituals. The relatives will not allow the procedure if it will delay the burial arrangements.

Several participants expressed negative perception towards conventional biopsy because it causes disfigurement of the face and body of the deceased. They preferred MITS because it keeps the facial figure. Some suggested that the procedure be introduced in adults also because it may be preferred compared to conventional autopsy. Some said that the many cuts made on the face makes it unsightly and unrecognizable to the extent that sometimes it is difficult to confirm whether you have been given the right body from the health facility.

*“In my opinion I support this system because if you get a challenge and they do a post-mortem, when they are back you cannot know if they’re the real person, but for this one if they just take off samples and test, you will remain with your face and you will not change. I am in support that this system should start immediately.”* (Participant 15, FGD men)

Some participants mentioned that they prefer to bury a complete body with no body parts missing. People believe that several organs are removed from the body during conventional autopsy and body is buried without the internal organs. MITS would be preferred because one will bury a complete body.

### Self-efficacy

This theme, which relates to the belief that one can do the intervention, in this case the MITS procedure, by the health system was most pronounced by the health workers. In short, the health workers believed they could learn and do the MITS procedure given sufficient training and support. It was mentioned that none of the health in the three health facilities had had training in MITS.

*“Yes [it can be done in my health facility], I think it can be done as long as we have the health workers who have been trained to do that.”* (Participant 3, IDI health worker).

## Discussion

In this study, we explored the perceptions of the community and healthcare workers about MITS. Although the results reflected mixed views about MITS, most of the participants felt that MITS could be a useful procedure and would be acceptable in this community, especially when accompanied with sensitization. The participants’ willingness to accept MITS were driven by the need to know the cause of stillbirth.

MITS was acceptable by most participants because it was perceived to confirm the cause of stillbirth hence leading to the prevention of future stillbirths, improved healthcare during labour and delivery, it would keep the infant’s body intact, it was believed to be less painful to the body, it is fast and keeps the facial figure of the body. The perceived barriers to implementation of MITS included religious beliefs such as the need to refrain from questioning God’s authority, fear of witchcraft, lack of trust in the health system and health facility readiness. To implement this programme, training of health workers and extensive community sensitization were suggested in addition to finding a solution to space (to perform the procedure) limitations and heavy workload among health workers.

In this study, the motivation to confirm the cause of death was a major driving factor in accepting MITS among the community and the health workers. This is similar to a qualitative study done in Mozambique to explore the acceptability of MITS in children [16,17]. Similar findings were also identified in a qualitative study done in Tanzania where participants wanted MITS to be done especially when the cause of death was uncertain [26]. In our study, the health workers also perceived that MITS would be important when they were not able to determine the cause of death through the routine investigations. The finding that MITS was seen as an avenue to prevent future stillbirths is similar to other studies done in Mozambique [17,19] that found that preventing future infections or inheritable diseases was a motivating factor to accepting MITS.

Confirming the cause of death through MITS was viewed as a potential route for acquitting suspected witchcraft. This finding is similar to the results of the qualitative study in Mozambique where MITS was perceived to save the elderly who would often be accused of causing the stillbirth through witchcraft [17].

MITS was perceived to be fast. People preferred a fast procedure because of the need to bury as soon as possible after birth. These findings are similar to other studies done in India and Mozambique [16,27]. The practice of burying a stillborn baby soon after birth has been previously reported in Uganda, Kenya and several African communities [28].

MITS was also perceived to be acceptable by most participants because the facial figure is not distorted. This is because they perceived that a face with several cuts and stitches is unsightly.

MITS was perceived to be acceptable because the body was kept intact. The perception that MITS may be more acceptable because it causes less damage is similar to results from a qualitative study done in Vietnam that found that because people consider the body sacred, they would prefer it not to be dissected but rather small hollow needles be used to access material for cause of death determination [29]. The same study also found that some people did not accept it because they considered that even removal of small portions of tissue would be unacceptable cause of the need to maintain an intact body [29].

The perception by health workers that MITS may be a problem to implement due to the already excess workload health workers have is a shared concern with previous studies in Vietnam where health workers were willing to implement MITS [29].

It was a common suggestion that sensitization of the community about MITS was necessary. This was mentioned by both the community participants and the health workers. This is similar to results from a qualitative study done in Vietnam where the health workers believed that mass sensitization would need to be done for to increase acceptability of MITS [29].

In this study, religious beliefs were cited as barriers to implementation of MITS. Driven by the need to bury an intact body so that it returns to the afterlife unmaimed, the participants preferred MITS over conventional autopsy. Because of the belief that there is a life after death, some participants opined that one should be buried with all organs intact so that they live a complete life without a maimed body. This opinion was shared by several religious participants. A study done in Ethiopia in a predominantly Muslim community found that religious participants did not want the body to be *mutilated* [30]*.*

The finding in our study that there was a perception that the body feels pain and MITS or autopsy is seen as a form of torture to the body is shared with findings from studies from other countries that found that MITS was viewed as causing pain, disrespectful, torturing, humiliating, or disturbing act against the deceased [19,30]. Furthermore, previous studies have found that Muslim culture does not accept the body to be dissected or left in hospital for long because it was perceived that the body is suffering [17].

In our study, religious beliefs provided mixed perceptions about MITS. Although several participants who were Muslims felt MITS should not be performed because it would be disrespectful or cause pain to the body, some believed that MITS was acceptable because of its minimal invasiveness and because of the need to find out the cause so as to avoid stillbirths in the same mother or other women in future. Previous studies have yielded varying results concerning the Islamic faith; while some studies have confirmed the Muslims tradition demands to have an intact body buried [26], some have found that MITS or other forms of autopsy are acceptable because they may save other lives in future [31].

Among the barriers identified in this study was the perception that some people did not see any value in performing the MITS because the child was already dead. This is similar to other studies that have found that some people do not see any value in MITS since the baby was already dead or because they believed that a baby is stillborn because it is the will of God [19].

Some participants believed that the conventional autopsy also involves ‘harvesting’ organs of the body to be donated for transplant. MITS was preferable because it did not involve removal of organs. This finding is similar to a study done in Tanzania where the community perceived that organs were removed either for donation to other people or sexual organs were used to enhance sexual prowess of other people after donation [26].

Thus, culturally sensitive approaches are needed when introducing new procedures. Some previous studies have used two counsellors and performed the procedures as the parents complete the discharge process of the health facility to fasten the process to reduce delays [32]. Obtaining evidence of permissibility of MITS from local religious leaders has also been done in previous studies. This may enhance acceptability among the Muslim communities [32].

The study had some limitations. We investigated the acceptability hypothetically without implementing the procedure through training. The opinion of the same participants could change when the procedure is introduced. This study was done in an area that is predominantly occupied by people from one ethnic group. The findings are believed to be representative for this ethnic group, but acceptability would need to be assessed for different regions and cultures in Uganda given implementation or roll-out. That said, we believe training and sensitisation are universal public health measures necessary irrespective of culture.

The study had several strengths. The study involved the community, the leaders and health workers. This ensured that triangulation of the findings across informant groups.

## Conclusion

Despite several cultural and religious beliefs being barriers to the implementation of MITS in Mbale, eastern Uganda, MITS is likely to be acceptable given sufficient training and sensitisation. Based on the statements from health staff, it also seems to be welcomed and feasible with future advantages including improved knowledge and practices and better preventive measures in the future. Sensitization of community members about biomedical causes of stillbirths and the need for MITS to confirm the cause of death could reduce stigma associated with accusation and witchcraft. We recommend that innovative culturally sensitive methods should be used to improve acceptability of the procedure.

## Supporting information

S1 Fig Photographs of sampling done in minimally invasive tissue sampling procedure. (TIF)

S2 Fig Inclusivity in global research questionnaire. (DOCX)
