# Supplementary figures and images for: Acceptability of minimal invasive tissue sampling (MITS) for stillbirths in Eastern Uganda
Source: PLoS One. 2025 Oct 13;20(10):e0334548. doi: 10.1371/journal.pone.0334548 (PMC12517472; doi:10.1371/journal.pone.0334548)

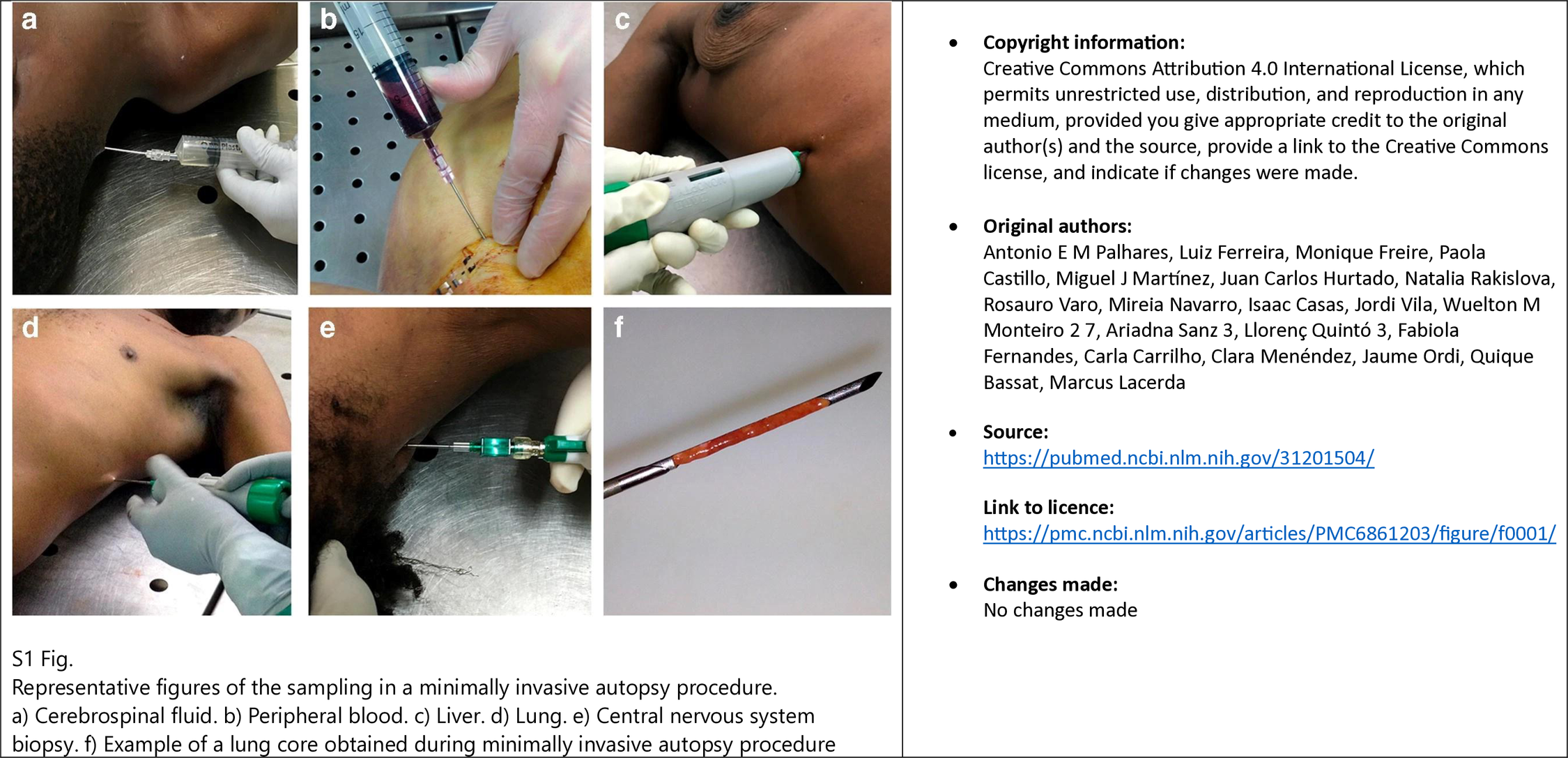

Supplement: S1 Fig — (TIF) [file pone.0334548.s001.tif]
